# Estimating racial differences in risk for CLABSI in a large urban healthcare system

**DOI:** 10.1017/ash.2023.292

**Published:** 2023-09-29

**Authors:** Giancarlo Licitra, Scott Fridkin, Zanthia Wiley, Lindsey Gottlieb, William Dube, Vishnu Ravi Kumar, Rachel Patzer, Radhika Prakash Asrani

## Abstract

**Background:** Socioeconomic barriers or divergent implementation of prevention measures may impact risk of healthcare-associated infections by racial groups. We utilized a previously studied cohort of patients to quantify disparities in central-line–associated bloodstream infection (CLABSI) risk by race accounting for inherent differences in risk related to device utilization. **Methods:** In a retrospective cohort of adult patients at 4 hospitals (range, 110–733 beds) from 2012 to 2017, we linked central-line data to patient encounter data: race, age, comorbidities, total parenteral nutrition (TPN), chemotherapy, CLABSI. Analysis was limited to patients with >2 central-line days and <3 concurrent central lines. Patient exposures were calculated for each central-line episode (defined by insertion and removal dates); analysis of central-line episode-specific risk of CLABSI among Black versus White patients adjusted for clinical factors, duration of central-line episode, and central-line risk category (ie, low: single port, dialysis or PICC; medium: single temporary or nontunneled; or high: any concurrent central-lines) in Cox proportional hazards regression of time to CLABSI. **Results:** In total, 526 CLABSIs occurred a median of 14 days after insertion among 57,642 central-line episodes in 32,925 patients. CLABSIs occurred in similar frequency across racial groups: 217 (1.7%) among Black patients, 256 (1.6%) among White patients, and 11 (1.6%) among Hispanic patients (also 42 among unknown or other race). Duration of central-line episode was similar between racial groups (median, 5 days). Black patients were less likely to have medium-risk central lines (34%) compared to white patients (RR, 0.82; 95% CI, 0.79–0.84), but they had a similar frequency of high-risk central lines (21%; RR, 1.0; 95% CI, 1.0–1.1). Compared with low-risk central lines, risk of CLABSI was increased among medium-risk central lines (RR, 1.3; 95% CI, 1.0–1.7) and high-risk central lines (RR, 2.2; 95% CI, 1.8–2.7). CLABSIs were more likely in TPN central lines (RR, 2.3; 95% CI, 1.9–2.7) than others, but they were not more likely among Black patients than White patients (RR, 0.9; 95% CI, 0.1–1.1). In survival analysis, there were 24,700 central-line episodes among Black patients compared to 26,648 episodes among White patients; adjusting for central-line risk and TPN, the risk of CLABSI was similar during the first 21 days of central-line use (adjusted hazard ratio, 1.08; 95% CI, 0.88–01.32) (Fig. 1). **Conclusions:** After accounting for central-line configuration, Black patients did not have a higher risk of CLABSI within 21 central-line days. Further evaluation is warranted to assess racial disparities in risks of other healthcare-associated infections and to determine whether a lack of CLABSI-specific racial disparities can be replicated in other regions and healthcare systems.

**Figure:**
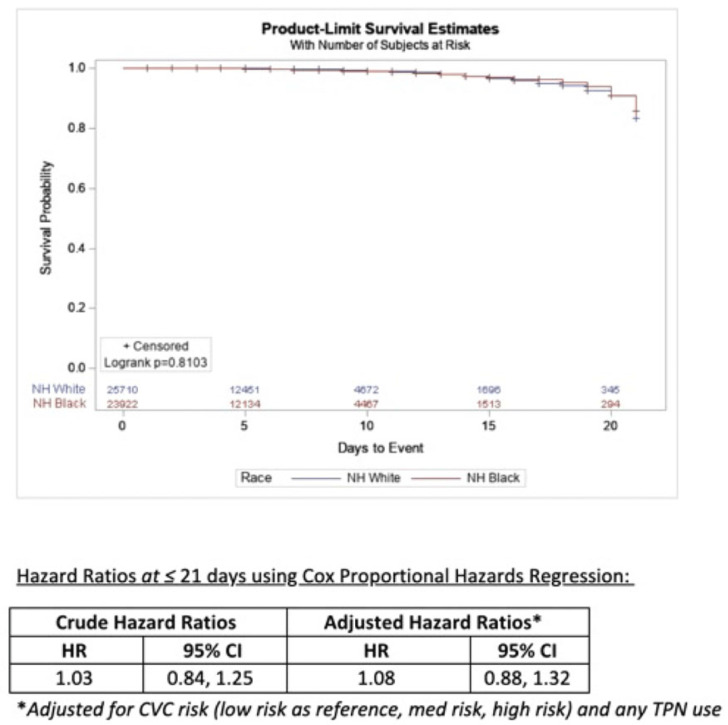

**Disclosures:** None

